# Parathyroid Retrospective Analysis of Neoplasms Incidence (pTRANI Study): An Italian Multicenter Study on Parathyroid Carcinoma and Atypical Parathyroid Tumour

**DOI:** 10.3390/jcm12196297

**Published:** 2023-09-29

**Authors:** Angela Gurrado, Alessandro Pasculli, Nicola Avenia, Rocco Bellantone, Marco Boniardi, Isabella Merante Boschin, Pietro Giorgio Calò, Michele Camandona, Giuseppe Cavallaro, Fabio Cianchi, Giovanni Conzo, Vito D’Andrea, Carmela De Crea, Loredana De Pasquale, Paolo Del Rio, Giovanna Di Meo, Gianlorenzo Dionigi, Chiara Dobrinja, Giovanni Docimo, Fausto Famà, Attilio Galimberti, Laura Giacomelli, Giuseppa Graceffa, Maurizio Iacobone, Nadia Innaro, Celestino Pio Lombardi, Gabriele Materazzi, Fabio Medas, Barbara Mullineris, Luigi Oragano, Nicola Palestini, Giuliano Perigli, Angela Pezzolla, Francesco Paolo Prete, Marco Raffaelli, Giuseppina Renzulli, Lodovico Rosato, Gregorio Scerrino, Lucia Ilaria Sgaramella, Salvatore Sorrenti, Carlotta Testini, Massimiliano Veroux, Guido Gasparri, Mario Testini, pTRANI Study Group

**Affiliations:** 1 Department of Precision and Regenerative Medicine and Ionian Area (DiMePre-J), University Medical School of Bari, 70124 Bari, Italy; angela.gurrado@uniba.it (A.G.); dimeojenny@gmail.com (G.D.M.); angela.pezzolla@uniba.it (A.P.); pretef@gmail.com (F.P.P.); ilaria.sgaram@gmail.com (L.I.S.); mario.testini@uniba.it (M.T.); 2 General and Endocrine Surgery Unit, S. Maria University Hospital, University of Perugia, 05100 Terni, Italy; nicola.avenia@unipg.it; 3 Division of Endocrine and Metabolic Surgery, Fondazione Policlinico Universitario “Agostino Gemelli” IRCCS, 00168 Rome, Italy; rocco.bellantone@unicatt.it; 4 General Oncology and Mini-Invasive Surgery Department, ASST Grande Ospedale Metropolitano Niguarda, 20162 Milan, Italy; mboniardi@gmail.com; 5 Endocrine Surgery Unit, Department of Surgery, Oncology and Gastroenterology, University of Padua, 35143 Padua, Italy; isabella.meranteboschin@unipd.it (I.M.B.); maurizio.iacobone@unipd.it (M.I.); 6 Department of Surgical Sciences, University of Cagliari, 09124 Cagliari, Italy; pgcalo@unica.it (P.G.C.); fabio.medas@unica.it (F.M.); 7 Department of Surgical Sciences, Molinette Hospital, University of Turin, 10126 Turin, Italy; michele.camandona@unito.it (M.C.); gasparri.guido@gmail.com (G.G.); 8 Department of Medico-Surgical Sciences and Biotechnologies, Sapienza University, 00185 Rome, Italy; giuseppe.cavallaro@uniroma1.it; 9 Digestive Surgery Unit, Department of Experimental and Clinical Medicine, University of Florence, 50134 Florence, Italy; fabio.cianchi@unifi.it (F.C.); giuliano.perigli@unifi.it (G.P.); testinicarlotta@gmail.com (C.T.); 10 Division of General, Oncological, Mini-Invasive and Obesity Surgery, Department of Medical and Traslational Sciences, University of Campania “Luigi Vanvitelli”, 80138 Naples, Italy; giovanni.conzo@unicampania.it; 11 Department of Surgical Sciences, Sapienza University, 00185 Rome, Italy; vitodandrea@uniroma1.it; 12 UOC Chirurgia Endocrina e Metabolica, Fondazione Policlinico Universitario “Agostino Gemelli” IRCCS, 00168 Rome, Italy; carmela.decrea@rm.unicatt.it (C.D.C.); marco.raffaelli@unicatt.it (M.R.); 13 Centro di Ricerca in Chirurgia delle Ghiandole Endocrine e dell’Obesità, Università Cattolica del Sacro Cuore, 00168 Rome, Italy; 14 Head and Neck Surgery Department, San Paolo Hospital, 20142 Milan, Italy; loredana.depasquale@ao-sanpaolo.it; 15 Department of General and Specialist Surgery, Parma University Hospital, University of Parma, 43126 Parma, Italy; paolo.delrio@unipr.it; 16 Division of Surgery, Istituto Auxologico Italiano IRCCS, 20122 Milan, Italy; gianlorenzo.dionigi@unimi.it; 17 Department of Pathophysiology and Transplantation, University of Milan, 20122 Milan, Italy; 18 Department of Medical, Surgical and Health Sciences, University of Trieste, 34125 Trieste, Italy; ch_dobrinja@yahoo.it; 19 Department of Advanced Medical and Surgical Sciences, University of Campania L. Vanvitelli, 80138 Naples, Italy; giovanni.docimo@unina2.it; 20 Department of Human Pathology in Adulthood and Childhood “G. Barresi”, University Hospital “G. Martino”, 98125 Messina, Italy; ffama@unime.it; 21 Department of Surgery, Legnano Hospital, 20025 Legnano, Italy; attilio.galimberti@asst-ovestmi.it; 22 Department of General and Speciality Surgery, Sapienza University, 00185 Rome, Italy; laura.giacomelli@uniroma1.it; 23 Department of Surgical Oncological and Oral Sciences, University of Palermo, 90127 Palermo, Italy; giuseppa.graceffa@unipa.it (G.G.); gregorio.scerrino@tiscali.it (G.S.); 24 Unit of Endocrine Surgery, AOU “Dulbecco”, University “Magna Graecia” of Catanzaro, 88100 Catanzaro, Italy; nadia.innaro@tiscali.it; 25 Endocrine Surgery Unit, Department of Surgical, Medical and Molecular Pathology and Critical Area, University Hospital of Pisa, 56121 Pisa, Italy; gabriele.materazzi@unipi.it; 26 Unit of General Surgery, Emergency and New Technologies, Modena Hospital, 41126 Modena, Italy; mullinerisbarbara@gmail.com; 27 Surgical Unit of General Surgery, “San Biagio” Hospital, 28845 Domodossola, Italy; luigioragano@libero.it; 28 Candiolo Cancer Institute, Fondazione Piemontese per l’Oncologia, 10060 Candiolo, Italy; nicolapalestini.doc@gmail.com; 29 Unit of Pathology, Department of Precision and Regenerative Medicine and Ionian Area (DiMePre-J), University Medical School of Bari, 70124 Bari, Italy; renzullipina@gmail.com; 30 Department of Surgery-ASL TO4, Ivrea Hospital, 10015 Ivrea, Italy; giordano.rosato@gmail.com; 31 Department of Surgery, Sapienza University, 00185 Rome, Italy; salvatore.sorrenti@uniroma1.it; 32 Department of Medical and Surgical Sciences and Advanced Technologies, University of Catania, 95100 Catania, Italy; veroux@unict.it

**Keywords:** parathyroid carcinoma, atypical thyroid tumour, parathyroidectomy, hyperparathyroidism, 2022 WHO criteria

## Abstract

Background: Parathyroid cancer (PC) is a rare sporadic or hereditary malignancy whose histologic features were redefined with the 2022 WHO classification. A total of 24 Italian institutions designed this multicenter study to specify PC incidence, describe its clinical, functional, and imaging characteristics and improve its differentiation from the atypical parathyroid tumour (APT). Methods: All relevant information was collected about PC and APT patients treated between 2009 and 2021. Results: Among 8361 parathyroidectomies, 351 patients (mean age 59.0 ± 14.5; F = 210, 59.8%) were divided into the APT (n = 226, 2.8%) and PC group (n = 125, 1.5%). PC showed significantly higher rates (*p* < 0.05) of bone involvement, abdominal, and neurological symptoms than APT (48.8% vs. 35.0%, 17.6% vs. 7.1%, 13.6% vs. 5.3%, respectively). Ultrasound (US) diameter >3 cm (30.9% vs. 19.3%, *p* = 0.049) was significantly more common in the PC. A significantly higher frequency of local recurrences was observed in the PC (8.0% vs. 2.7%, *p* = 0.022). Mortality due to consequences of cancer or uncontrolled hyperparathyroidism was 3.3%. Conclusions: Symptomatic hyperparathyroidism, high PTH and albumin-corrected serum calcium values, and a US diameter >3 cm may be considered features differentiating PC from APT. 2022 WHO criteria did not impact the diagnosis.

## 1. Introduction

Parathyroid cancer (PC) is a very uncommon malignancy with an incidence of 0.5–5% of primary hyperparathyroidism (PHPT) cases, without predominance in both genders, and usually diagnosed in the fifth decade of life [1,2,3,4].

This malignancy is frequently sporadic, but it may be part of the hyperparathyroidism-jaw tumour syndrome (HPT-JT) or the multiple endocrine neoplasia types 1 (MEN1) and 2A (MEN2A) [5,6]. HPT-JT syndrome shows germline mutations of the CDC73 gene (located in 1q32.1). In contrast, a somatic mutation of the same gene is sporadic in 70% of sporadic PC; however, up to 33% of PC are only apparently sporadic because they present a germline mutation, constituting a variant of the HPT-JT syndrome [2,6]. CDC73 gene encodes for the parafibromin (tumour suppressor protein, PFIB), and the mutations result in a loss of PFIB expression. The 2022 World Health Organization (WHO) classification of parathyroid tumours defined the cases of nuclear PFIB deficiency as “parafibromin deficient parathyroid neoplasm” because they could apply to every parathyroid disease [7]. On the other hand, the persistence of PFIB expression does not exclude the possibility of underlying CDC73 inactivation as point mutations of the gene [8].

Clinically, most patients affected by PC have a history of nephrolithiasis, nephrocalcinosis, skeletal involvement and symptoms due to hypercalcemia (i.e., polyuria, polydipsia, weakness, anorexia, vomiting, weight loss, confusion); sometimes a palpable cervical mass is associated; rarely nodal or distant metastases are reported at diagnosis. Laboratory tests generally show hypercalcemia (>13 mg/dL) and high levels of parathormone (PTH) (2–10 times above the normal range); <10% of cases are normocalcemic. Potentially life-threatening is the hypercalcemic crisis, associated with renal failure, cardiac arrhythmia, or reduced consciousness until coma [9]. Moreover, a serum alkaline phosphatase level of at least 285 IU/L and a lesion size greater than 3 cm have been related to suspicion of PC [1,10].

Ultrasonography (US) and MIBI (technetium 99 m sestamibi scintigraphy) are the most used imaging studies for detecting parathyroid abnormalities in patients with PHPT. Fine needle aspiration biopsy (FNAB) is not recommended when PC is suspected because cytology is ineffective in distinguishing malignancy and because of the risk of spreading neoplastic cells [1,11]. In contrast, the FNAB is safe and effective in the metastatic setting, usually with a PTH aspirate-hormone test that confirms PTH-secreting tissue. Computed tomography (CT) and magnetic resonance (MR) scans help detect the parathyroid mass and infiltration of surrounding structures and distant metastases. Positron emission tomography (PET)/CT helps stage loco-regional and distant metastatic, recurrence, or residual neck disease. 18-fluoro choline-PET (^18^F-Choline-PET) is a promising diagnostic technique for the pre-operative localisation of parathyroid lesions [12]. However, PC diagnosis is often post-operative and sometimes post-recurrence due to the challenge of distinguishing malignancy from adenoma or multiglandular parathyroid disease [2,6].

Macroscopically, PC is often a sizeable stony-hard mass that tends to adhere to or invade the surrounding structures. Histologic features were defined first in 1973 by Schantz and Castleman [13] and afterwards redefined until the current 2022 WHO classification [7] that confirms the diagnosis of PC with unequivocal evidence of microscopic invasion or distant metastases, as already defined in the 2017 WHO classification [14]. Neoplastic invasion may be infiltration into adjacent neck structures, angioinvasion, or perineural or lymphatic invasion. The coexistence of several features among cellular nets in a thickened connective tissue, tumour cells in the capsule, adherence to adjacent structures without frank invasion, band-like fibrosis, trabecular growth, above five mitoses per 50 high-power fields/per 10 mm^2^, atypical mitotic figures, coagulative necrosis, PFIB loss, Ki-67 labelling index >5%, but lacking evidence of capsular, vascular, or perineural invasion or distant metastases, are called the atypical parathyroid tumour (APT). APT in 2022 WHO nomenclature has replaced the previous atypical parathyroid adenoma definition (APA) to reflect the uncertainty of the malignant potential of this entity [7]. The Schulte b classification divides the PC into low risk (capsular invasion or invasion of surrounding soft tissues) or high risk (vascular invasion, lymph node metastases, invasion of vital organs, or distant metastases) [15].

Complete surgical resection with clear margins and without capsular rupture and spillage of neoplastic cells at the first operation is the gold standard for treatment to avoid local recurrence [1]. To obtain clear margins, the treatment should consist of *en-bloc* resection of the tumour with the ipsilateral thyroid lobe without compromising the tumour capsule and adjacent structures [1]. Some authors suggest routine dissection of the ipsilateral central compartment [2,16,17], but there is no evidence that prophylactic neck dissection improves survival.

After surgery, follow-up aims to detect loco-regional recurrence or metastases by closely monitoring serum calcium and PTH (i.e., bi-annually for five years and then yearly) and performing neck ultrasounds annually [3]. When PC diagnosis is post-operative, Xue et al. showed that the recurrence rate was reduced by reoperation (ipsilateral lobectomy and central compartment lymph nodes dissection) within one month [16]. However, other authors suggest close follow-up for patients with complete initial resection and if serum calcium and PTH levels are normalised [3,18]. Indeed, patients often develop distant metastases or recurrent disease, and a frequent cause of death is uncontrolled severe hypercalcemia [4,15,16]. Often, multiple surgical interventions are performed over time in order not only to remove the tumour but also to control the PTH-driven hypercalcemia that represents the primary cause of morbidity and mortality [3]. PC usually presents an indolent course, and the long-term survival is favourable: 78–91% and 60–72% overall survival at five and ten years, respectively [1]. Calcimimetics, denosumab and bisphosphonates are the cornerstones of medical treatment. The role of radiation therapy is debated because of PC radioresistance, while cytotoxic chemotherapy is ineffective [1,2,3,19,20]

In December 2019, 24 Italian institutions affiliated with the Unitarian Society of Italian Endocrine Surgery (SIUEC) established the Italian Parathyroid Carcinoma Study Group (IPCSG) to collect, share, analyse, and review all the information available on PC and APT. This study is a retrospective analysis of parathyroid neoplasm incidence, called pTRANI (paraThyroid Retrospective Analysis of Neoplasms Incidence), because the IPCSG first met in the Italian town of Trani in 2019. The study aims to specify PC incidence among parathyroidectomies, deepen the knowledge of this disease’s peculiar clinical, functional, and imaging characteristics, and improve its differentiation from APT. In addition, this study evaluates the possible changes in the distribution of APT and PC after introducing the 2022 WHO criteria.

## 2. Materials and Methods

The protocol of this retrospective multicenter study received the approval of the Ethics Committees of all participating centres. The manuscript has been structured according to the STROBE (Strengthening the Reporting of Observational Studies in Epidemiology) checklist for observational studies [21]. Data were obtained through a form sent to the 24 Italian Institutions part of the IPCSG, collecting all relevant information about PC and APT patients treated between 1 January 2009, and 31 December 2021. Follow-up was updated until 30 June 2022. Inclusion criteria were histopathologic diagnosis of PC or APT established by a pathologist using the 2004 and 2022 WHO criteria and a follow-up period of at least six months.

The database recorded pre-operative variables (demographic data, symptoms and signs, pre-operative diagnosis and localisation of parathyroid disease, pre-operative biochemistry), operative variables (type of surgery, intrao-perative features, and operative time), post-operative and follow-up variables (histology, biochemistry, complications, length of hospitalisation, staging, persistence, progression or recurrence at 12 months, mortality).

Pre-operative workup included albumin-corrected serum calcium, inorganic phosphorus, intact PTH, 25-hydroxy-vitamin D, alkaline phosphatase, 24-h urinary calcium and phosphorus. Localisation studies were US colour-Doppler of the neck and MIBI scan in most patients, whereas computed tomography, nuclear magnetic resonance, single photon emission computed tomography (SPECT)/CT and PET/TC were used in selected cases.

The surgical approach consisted of a cervicotomy for bilateral neck exploration, multiglandular disease, recurrences, pre-operative or intra-operative evidence of macroscopical infiltration of surrounding structures, and the need for lymph node dissection. In the case of single parathyroid involvement and precise pre-operative localisation, a minimally invasive video-assisted parathyroidectomy (MIVAP) was performed. A sternotomy or robotic approach was performed for ectopic affected parathyroid. Intra-operative PTH assay, reviewed adopting the Rome criteria, neuromonitoring and drainage were used according to each centre practice.

The histological diagnosis was recorded as performed soon after surgery and reviewed based on the 2022 WHO criteria. When available, immunohistochemistry about PFIB and Galectin-3 (GAL-3) was recorded. Risk stratification according to Schulte b classification was obtained from pathological reports.

Two independent reviewers checked all source data (N.A., G.G.). Missing values were handled through listwise deletion. Patients who died of other causes were excluded to reduce survival study bias. 

Continuous variables were compared with the Student’s t-test for independent samples. Pearson’s χ^2^ test or Fisher’s exact test were used for frequencies when appropriate. Wilcoxon signed-rank test was used for paired data comparisons. A multivariate analysis was performed using logistic regression to relate significantly different outcomes. *p* < 0.05 was considered statistically significant. The analyses reported were performed with Stata 14 (StataCorp LSJ, College Station, TX, USA).

## 3. Results

### 3.1. Patient Characteristics

The study population consisted of 8361 patients who had undergone parathyroidectomy. Of these, 351 patients (4.2%; mean age: 59.0 ± 14.5; range: 16–88 years; F = 210, 59.8%) were divided into the APT group (N = 226, 2.7%) and the PC group (N = 125, 1.5%).

Table 1 summarises demographic data, risk factors, and symptoms.

PC group showed significantly higher rates (*p* < 0.05) of men affected, concomitant thyroid disease, bone involvement, and abdominal, cardiovascular and neurological symptoms than the APT group (50.4% vs. 35.0%, 7.2% vs. 1.3%, 48.8% vs. 35.0%, 17.6% vs. 7.1%, 4.0% vs. 0.4% and 13.6% vs. 5.3%, respectively). In patients with hereditary diseases, the mutational status was available for two patients with HPT-JT, showing CDC73 mutations c276delA p.Asp93Ilefs*16 and c.-2insG (g.5182insG), and one patient with MEN1, showing mutation of exon 3 c.467G > A.

### 3.2. Pre-Operative Data

The results of pre-operative blood and urinary tests are summarised in Table 2. 

Significantly (*p* < 0.05) higher values of pre-operative intact PTH (734.2 ± 741.0 pg/dL) and albumin-corrected calcium (12.6 ± 2.1 mg/dL) were evident in the PC group vs. APT one (509.8 ± 585.0 pg/dL and 12.1 ± 1.4 mg/dL, respectively), while values of inorganic phosphorus were lower (2.1 ± 0.8 mg/dL vs. 2.4 ± 1.1 mg/dL, *p* < 0.05).

During the pre-operative workup, ultrasound (US) colour-Doppler of the neck was performed in 341 patients (97.2%), in association with technetium 99 m sestamibi scan in 266 (75.8%). In addition, computed tomography (CT) scan of the neck and chest was used in 67 (19.1%), magnetic resonance (MR) imaging of the neck in 12 (3.4%), positron emission tomography (PET)/CT in 11 (3.1%), and single photon emission computed tomography (SPECT)/CT scan in 47 (13.3%) patients.

Table 3 shows the data from pre-operative imaging studies. 

A statistically significant difference between the PC vs. APT group was evidenced in terms of US-diameter > 3 cm (30.9% vs. 19.3%, *p* = 0.049) and suspected nodal metastases (5.8% vs. 0%, *p* < 0.01). In contrast, between-group differences for concordance of localisation, suspected infiltration of surrounding structures and distant metastases were not significant. Suspected surrounding organs infiltrated at pre-operative imaging (US or CT scan) were the thyroid in four (1.1%) patients, the oesophagus in three (0.9%), the internal jugular vein in one (0.3%), and the trachea in one patient (0.3%). Suspected distant metastase localisations were bones in three patients (0.9%), the liver in one (0.3%), and the pancreas in one (0.3%). Overall, selecting complete data from intra-operative surgical records, US and MIBI sensitivity in localising APT were, respectively, 84.4% and 84.3%, while for PC, they resulted in 92.7% and 83.6%.

To predict the histological diagnosis of PC and APT by combining the gender distribution, bone involvement, values of pre-operative albumin-corrected serum calcium and serum intact PTH with US diameter > 3 cm, a logistic regression model was built. A significant effect of calcium values was demonstrated (Table 4).

### 3.3. Intra-Operative Data and Histological Features 

Cervicotomy was the most common surgical approach (303 cases, 86.3%), followed by MIVAP (46 cases, 13.1%). One patient (0.3%) underwent sternotomy, and one was treated with a robotic approach (0.3%). Table 5 depicts the details of operative data. 

No significant differences were evident in the surgical approach comparing APT and PC groups. However, ipsilateral one-stage removal of normal parathyroid gland was significantly more common in the PC than in the APT group (19.2% vs. 10.6%, *p* = 0.009), as well as ipsilateral hemithyroidectomy (44.0% vs. 18.1%, *p* < 0.001) and central neck dissection (16.8% vs. 5.8%, *p* < 0.001). Total thyroidectomy was associated with parathyroid surgery in 8.0% of PC and 7.1% of APT (*p* < 0.001).

Resection of surrounding organs was more common in the PC than in the APT group (8.0% vs. 1.8%, *p* = 0.018). Surrounding organs resected were the thymus in seven patients (2.0%), oesophagus in three (0.9%), strap muscles in two (0.6%), and recurrent laryngeal nerve in two (0.6%).

Complete information about intra-operative parathormone (ioPTH) determination was available in 112 (32.0%) patients, of whom 102 (91%) showed a significant reduction after the removal of the pathological gland according to the Rome criterion. Intra-operative neuro-monitoring (IONM) was used in 57 (16.2%) patients, and a drain was placed in 198 (56.4%). No patient underwent synchronous treatment of metastases. Operative time was significantly shorter in the APT group than in the PC group (71.9 ± 46.0 vs. 105.1 ± 63.5 min, *p* < 0.001). Overall, post-operative hospital stay was significantly longer in the PC group than in the APT group (3.2 ± 1.1 vs. 2.2 ± 0.9, *p* < 0.001). Albumin-corrected serum calcium at discharge showed no significant differences between APT and PC (9.3 ± 1.0 vs. 9.5 ± 1.2 mg/dL, *p* = 0.190), neither did the PTH values (47.7 ± 115.0 vs. 47.5 ± 81.5 pg/dL, *p* = 0.987). 

Considering only the cases treated before 2017, Table 6 shows the changes in microscopic diagnosis after revision according to 2022 WHO criteria. 

Of the 210 patients treated before 2017 (59.8%), there was a non-significant increase in patients with APT (66.7% vs. 64.3%). According to Schulte b classification, there were 39 patients at high risk in the PC group (31.2%).

### 3.4. Post-Operative Data and Follow-Up

Post-operative complications are in Table 7: in the PC group, there was a significantly (*p* < 0.05) higher frequency of transient hypoparathyroidism (33.3% vs. 22.3%) and permanent recurrent laryngeal nerve palsy (8.3% vs. 1.4%).

The mean follow-up time was 44.6 ± 38.6 months. After surgery, one patient with PC (0.8%) underwent radiotherapy; 13 patients, with no significant differences between APT and PC group (2.7% vs. 5.6%), were on calcimimetics therapy. During the first 12 months of follow-up, three cases of persistence (0.4% vs. 1.6%), seven of progression (1.8% vs. 2.4%) and five of recurrence (0.9% vs. 2.4%), with no significant differences between APT and PC, were recorded.

During the entire follow-up, a significantly higher frequency of local recurrences was observed in the PC than in the APT group (N = 10, 8.0% vs. N = 6, 2.7%, *p* = 0.022). Three patients in the PC group (2.4%) discovered distant metastases (bones in one patient, liver in one patient, lung in one patient). Re-surgery for local recurrences or distant metastases was performed in 17 patients (4.8%). In the PC group, two patients (1.6%) underwent chemotherapy (capecitabine and temozolomide), one radiotherapy (0.8%), and two (1.6%) immunotherapy (denosumab). Among PC patients, excluding deaths for other reasons (three patients), mortality due to consequences of cancer or uncontrolled hyperparathyroidism was 3.3% (four patients). Ninety-three patients (26.5%) were lost to follow-up.

## 4. Discussion

PC is a very rare malignant endocrine neoplasm present with the clinical manifestations of the PHPT, and those from the tumour burden only belatedly, due to an indolent tendency to local invasion. APT, previously called APA, demonstrates atypical cytological and architectural features similar to PC but does not show the unequivocal capsular, vascular or perineural invasion or infiltration into surrounding structures. The literature lacks a large, helpful series to define the incidence of PC and APT after parathyroid surgery and to analyse these diseases’ diagnostic and prognostic features. Therefore, the IPCSG designed this multicenter retrospective study to specify PC and APT incidences among parathyroidectomies before and after introducing 2017 WHO criteria, revised in 2022, and to deepen the knowledge of these forms of PHPT in terms of clinical, imaging and prognostic characteristics.

In this large series, PCs showed a prevalence of 1.5%, similar to previous reports [2,3,15,16,22,23]. The prevalence of men, patients with concomitant thyroid disorders, patients with symptoms concerning bone involvement, abdominal, cardiovascular and neurological symptoms, and values of pre-operative intact PTH and albumin-corrected calcium were higher in the PC group than in the APT one. PC was very rarely associated with a familial history (0.6% of cases in the contest of HPT-JT syndrome) and with a Multiple Endocrine Neoplasia type 1 (MEN1) syndrome (0.3% of cases in MEN1). HPT-JT is a hereditary autosomal dominant disorder with variable and incomplete penetrance with a 15–20% risk of developing PC [24]; thus, germline CDC73 mutation testing should be recommended for all patients with PC. MEN1 is an autosomal dominant hereditary syndrome with high penetrance that more frequently includes parathyroid adenoma and very uncommonly PC [25].

No consensus currently exists on the pre-operative parathyroid imaging algorithm [26]. Regarding pre-operative localisation studies, the US of the neck was the most used, followed by technetium 99 m sestamibi scan, as reported in the literature [1,2,27,28]. The US seem to be very useful in localising enlarged parathyroid glands, and the identification of lesions of considerable size (>3.0 cm) with marginal irregularity due to local tissue invasion, heterogeneous echotexture, and calcifications is suggestive of malignancy [29]. Following this literature evidence [29,30], the reported data showed a higher prevalence of diameter >3 cm for PCs than APTs using ultrasound. The literature reported that in differentiating PC and PA, the US has a sensitivity of 100%, specificity of 96.9%, and accuracy of 97.4% [30]. A study calculated that the ratio between the depth and width of the lesions (D/W ratio) correlates with the probability of malignancy, and a D/W ratio > 1 was considered suggestive of malignancy [31]. Moreover, the US evidence of nodal metastases was peculiar to the PC group, and this finding should raise suspicion of malignancy during pre-operative workup. Instead, no statistical differences were reported about the concordance of localisation, suspected infiltration of surrounding structures and suspected distant metastases between PC and APT groups; however, the rarity could have limited the relevance of this potential tool. Until now, the technetium 99 m sestamibi scan was not considered adequate to differentiate between malignant and benign parathyroid lesions. However, the peak of retention index (Ripeak) of MIBI has recently been correlated to a pre-operative differential diagnosis of PC [32]. A CT scan showing a mass with a high short-to-long axis ratio, irregular shape, peritumoral infiltration, calcification, and low contrast enhancement may identify a PC [33]. On MR, all parathyroid lesions are very bright on fat-saturated T2W images, and PC are also large, ill-defined and very heterogeneous on MR, including DWI [34]. Of note was the study of Christakis et al. [35] that compares the accuracy of US, CT and MIBI, either alone or in combination and concludes that the sensitivity of every single pre-operative procedure was approximately 80% and reached 95% or more when the three methods were used together. Recently, positron emission tomography (PET) using the positron-labelled choline analogue 18F-fluoro choline (FCH-PET) has demonstrated high accuracy in the detection of benign parathyroid lesions, especially when other modalities are negative or discordant [36,37,38]. FCH PET and SPECT/CT mainly help patients undergoing reoperations [39]. However, these features are not specific to distinguish PC from APT in the pre-operative setting, and the challenge to the clinicians remains.

Complete and oncologic surgical resection with clear margins for PC or suspicious PC is the treatment that could affect long-term outcomes for the patient regarding local relapse and mortality [1,4,15,16,28]. The minimally invasive approach should be the standard for PHPT treatment, but most PC patients are treated through cervicotomy. A possible explanation may be that the decision on an adequate surgical procedure depends upon the pre-operative diagnosis of PC or benign lesion. If this is lacking, the cervicotomy could be more helpful. At surgery, if there is an intra-operative observation of a firm, adherent and invasive parathyroid mass, an *en-bloc* resection with surrounding structures (thyroid lobe) should be performed, and the surgical approach (cervicotomy, MIVAP or others) should be the one that seems to be more effective to avoid gland rupture and tumour spillage. These patients’ history is indeed marked by local recurrence [28]. No consensus exists regarding prophylactic neck dissection [2,16,17], whereas involved lymph nodes must be removed. The central neck dissection in one or two stages was more frequent in the PC group than in the APT one, confirming that the prophylactic neck dissection should not be performed. The occasional resection of neighbouring structures in the APT group could be explained with the recommendations to perform these *en-bloc* resections to avoid tumour rupture and spillage when PC is suspected [10]. In addition, when the PC diagnosis is post-operative, patients may undergo a completion surgery involving the ipsilateral parathyroid, thyroid lobe and central neck lymph nodes. In the literature, up to one-third of PCs seem to be macroscopically benign lesions [1,16]; additional surgery should be offered in a timely manner as a second chance for these patients. In this regard, the literature data are discordant because some authors [16] suggest ipsilateral hemithyroidectomy and central neck dissection within one month. In contrast, others [3,11,18] offer close follow-up for patients with complete initial resection. If the ioPTH assay during resection for the parathyroid lesion is very useful to confirm the disease identification, the effectiveness of the potential power to predict the recurrence or death of PC remains unknown. Only in a recent study was the decrease of more than 60% of PTH values correlated with prolonged recurrence-free survival on univariate analysis [28]. Intra-operative neuro-monitoring data are of limited value because of the few reported cases. However, we suggest the usefulness of IONM in high-risk bilateral neck exploration, especially in patients with associated thyrotoxicosis, ectopic (retrosternal) parathyroid lesions, suspected malignancy and in reinterventions [40], although the gold standard approach to preserve the RLNs is the accurate visual identification of the nerves [41].

The 2022 WHO classification distinguishes the parathyroid neoplasms in parathyroid adenoma (benign), APT (parathyroid tumour of uncertain malignant potential), and PC. Our study aimed to specify the incidence of PC among parathyroidectomies and verify if the modifications of the 2017 WHO classification could be considered more about terminology than contents. Histological criteria for pathological diagnosis of PC and APT (beyond the terminology) have not changed under the 2022 WHO classification [7]. The differential diagnosis of PC includes APT, parathyromatosis, a benign parathyroid adenoma/multiglandular disease with histologic alterations following FNAC or ethanol injection, and contour irregularities of the gland due to longstanding secondary/tertiary hyperparathyroidism. The clinical and laboratory information drives the differential diagnosis. An essential aspect of the revised classification is the suggestion to use ancillary immunohistochemical markers to differentiate between adenoma, APT and PC [7]. The adenoma often stains positive for PFIB and APC and negative for galectin-3 (GAL-3) and PGP9.5, and its Ki-67 index is often <1%. The APT, instead, stains positive or negative for the same immunohistochemical markers and shows a Ki-67 <5%. On the contrary, the PC is usually negatively stained for PFIB and APC and positive for GAL-3 and PGP9.5, with a Ki-67>5%. If the risk of recurrence and metastasis is specific to PC, APT may rarely be recurrent or metastatic, confirming that it has an uncertain malignant potential. However, a high risk of this behaviour seems to be proper of the APT with negative staining for PFIB [42], for whom a clinical and biochemical follow-up is recommended. Our results showed that the histologic revision of the cases treated before 2017, applying the 2017 and 2022 WHO criteria, showed no changes in histological diagnosis between PC and APT.

Complications after parathyroid surgery for PC are rarely reported. A higher prevalence of transient hypoparathyroidism and permanent recurrent laryngeal nerve palsy was observed in the PC group. The extensive surgery and cancer invasion of the laryngeal nerve can explain both results. In particular, laryngeal nerve palsy due to nerve resection for surgical radicality or infiltration by cancer during a lifespan is reported in up to 38% of cases [11,43,44]. After adequate surgical resection, further therapies are not standardised: no evidence of helpful adjuvant radiotherapy or chemotherapy is available [45,46]. PC is considered radioresistant; however, a recent report suggested the potential efficacy of adjuvant radiotherapy after radical surgery in maintaining adequately treated patients disease-free after a median follow-up of 12.5 years [47]. Since morbidity and mortality are mainly due to uncontrolled hypercalcemia, the pharmacotherapy of PC and APT with persistent/recurrent hypercalcemia is based on calcimimetics, whose role is well established in the literature [20,48,49]. In our series, the recurrence, persistence and progression rates of hypercalcemia after surgery during the first 12 months and the proportion of patients under calcimimetics therapy were not different between APT and PC cases. Although available literature data about these early outcomes are poor, Cetani et al. reported a recurrence rate of 3% after surgical resection of APT [50]. In comparison, PC shows a rate of up to 51% [1]. The recorded mortality of PC was 3.3%, with a mean follow-up of 44.6 ± 38.6 months. This is concordant with current survival rates reported by literature: 78–91% at five years [1].

This study has several limitations: its retrospective and multicentric nature were prone to selection bias and missing data. Moreover, this was not a survival study because of the different follow-up methods adopted by each centre and the high rate of loss to follow-up patients, so that no reliable conclusions could be made about prognostic factors. Despite these limitations, this is one of the most extensive series available in the literature. These data may help clear the epidemiological aspects of this rare disease, allowing us to underline clinical, functional, and imaging characteristics of high suspicion of parathyroid cancer.

## 5. Conclusions

Parathyroid carcinoma and atypical parathyroid tumours are rare diseases, accounting for 1.5% and 2.7% of parathyroidectomies. Symptomatic hyperparathyroidism, especially with bone involvement, high values of PTH and albumin-corrected serum calcium, and a US diameter of more than 3 cm, may be considered features of high suspicion of parathyroid carcinoma, differentiating it from the atypical parathyroid tumour. The introduction of the 2022 WHO criteria did not significantly modify the histological diagnosis. Further prospective multicentric studies may help to elucidate differences in prognostic factors of these two pathological entities.

## Figures and Tables

**Table 1:** Demographic data, risk factors, symptoms.

	APT N = 226 (64)	PC N = 125 (36)	*p* *
Age	58.2 ± 14.5	60.3 ± 14.2	0.205
Sex			
Male	79 (35.0)	63 (50.4)	0.009
Female	147 (65.0)	62 (49.6)	
Familiar history			
Cancer	20 (8.9)	11 (8.8)	0.987
FIHP, HPT-JT, MEN	3 (1.3)	4 (3.2)	0.252
Personal history			
Previous parathyroid surgery	8 (3.5)	7 (5.6)	0.412
Secondary/tertiary hyperparathyroidism	7 (3.1)	2 (1.6)	0.500
FIHP, HPT-JT, MEN	1 (0.4)	3 (2.4)	0.131
Cancer	16 (7.1)	13 (10.4)	0.279
Thyroid disorders	3 (1.3)	9 (7.2)	0.010
Symptoms			
Bone involvement (osteoporosis, osteopenia, brown tumours, fractures)	79 (35.0)	61 (48.8)	0.011
Kidney involvement (nephrolithiasis, nephrocalcinosis, chronic kidney failure)	91 (40.3)	57 (45.6)	0.332
Abdominal symptoms (nausea, pain, peptic ulcer, pancreatitis)	16 (7.1)	22 (17.6)	0.002
Palpable mass, compressive symptoms	4 (1.8)	4 (3.2)	0.462
Weight loss	5 (2.2)	8 (6.4)	0.073
Fatigue, weakness, joint pain	38 (16.8)	30 (24.0)	0.103
Cardiovascular symptoms	1 (0.4)	5 (4.0)	0.023
Neurological symptoms	12 (5.3)	17 (13.6)	0.009

Data are given as mean values ± standard deviations or absolute values with frequencies (calculated on available data) in brackets. APT atypical parathyroid tumour; PC parathyroid carcinoma; FIHP familial isolated hyperparathyroidism; HPT-JT hyperparathyroidism–jaw tumour syndrome; MEN multiple endocrine neoplasias. * Student’s *t*-test or Pearson’s χ^2^ test or Fisher’s exact test, when appropriate. *p* < 0.05 was considered statistically significant.

**Table 2:** Pre-operative blood and urinary tests.

	APT N = 226 (64)	PC N = 125 (36)	*p* *
Albumin-corrected serum calcium (mg/dL)	12.1 ± 1.4	12.6 ± 2.1	**0.003**
Serum intact PTH (pg/mL)	509.8 ± 585.0	734.2 ± 741.0	**0.003**
Serum inorganic phosphorus (mg/dL)	2.4 ± 1.1	2.1 ± 0.8	**0.023**
Serum 25-hydroxy vitamin D (ng/mL)	25.0 ± 27.5	26.1 ± 22.5	0.773
Serum Alkaline phosphatase (IU/L)	195.4 ± 281.5	225.6 ± 253.0	0.526
Urine 24 h calcium (mg)	368.8 ± 192.2	371.5 ± 234.3	0.938
Urine 24 h phosphorus (mg)	750.1 ± 448.8	716.3 ± 427.5	0.713

Data are given as mean values ± standard deviations. APT atypical parathyroid tumour; PC parathyroid carcinoma. * Student’s *t*-test. *p* < 0.005 was considered statistically significant.

**Table 3:** Pre-operative imaging results.

	APT N = 226 (64)	PC N = 125 (36)	*p* *
Diameter > 3 cm ^a^ (on 226 US examinations)	28 (19.3)	25 (30.9)	0.049
Concordance of localisation	209 (92.5)	113 (90.4)	0.498
Suspected infiltration of surrounding organs	2 (0.9)	5 (4.2)	0.054
Suspected neck nodal metastases	0 (0)	7 (5.8)	0.001
Suspected distant metastases	1 (0.5)	3 (2.5)	0.353

Data are given as mean values ± standard deviations or absolute values with frequencies (calculated on available data) in brackets. APT atypical parathyroid tumour; PC parathyroid carcinoma. ^a.^ Measured through ultrasound neck scan. * Student’s *t*-test or Pearson’s χ^2^ test or Fisher’s exact test, when appropriate. *p* < 0.05 was considered statistically significant.

**Table 4:** Logistic regression of pre-operative significant data to differentiate between APT and PC.

	Odds Ratio	Standard Error	z	95% Confidence Interval	*p* *
Sex	1.72	0.52	1.79	0.95–3.10	0.073
Bones involvement	1.78	0.54	1.88	0.98–3.22	0.060
Pre-operative albumin-corrected serum calcium	1.25	0.12	2.40	1.04–1.50	**0.016**
Pre-operative intact serum PTH	1.00	0.00	1.56	0.99–1.00	0.119
US diameter > 3 cm	1.52	0.52	1.22	0.77–2.98	0.221

APT atypical parathyroid tumour; PC parathyroid carcinoma. * Logistic regression. *p* < 0.05 was considered statistically significant. LR chi^2^(5) = 23.79; Prob > chi^2^ = 0.0002; Pseudo R^2^ = 0.0839.

**Table 5:** Operative data.

	APT N = 226 (64)	PC N = 125 (36)	*p* *
Surgical approach			0.162
Cervicotomy	192 (85.0)	111 (88.8)	
MIVAP	32 (14.2)	11 (8.8)	
Converted MIVAP	1 (0.4)	2 (1.6)	
Sternotomy	1 (0.4)	0 (0)	
Robotic	0 (0)	1 (0.8)	
Ipsilateral normal parathyroid removal			0.009
No	200 (88.5)	96 (76.8)	
Yes, in one stage	24 (10.6)	24 (19.2)	
Yes, after cancer diagnosis	0 (0)	2 (1.6)	
Surgery for recurrent disease	2 (0.9)	3 (2.4)	
Concomitant thyroid surgery			<0.001
No	167 (73.9)	57 (45.6)	
Ipsilateral HT in one stage	41 (18.1)	55 (44.0)	
Contralateral HT for thyroid disease	2 (0.9)	1 (0.8)	
Ipsilateral HT after cancer diagnosis	0 (0)	2 (1.6)	
Total thyroidectomy	16 (7.1)	10 (8.0)	
Central neck dissection			<0.001
No	213 (94.3)	102 (81.6)	
Yes, in one stage	13 (5.8)	21 (16.8)	
Yes, after cancer diagnosis	0 (0)	2 (1.6)	
Surrounding organ resection ^a^	4 (1.8)	10 (8.0)	0.018

Data are given as absolute values with frequencies (calculated on available data) in brackets. APT atypical parathyroid tumour; PC parathyroid carcinoma. MIVAP minimally-invasive video-assisted parathyroidectomy; HT hemithyroidectomy. ^a^ Esophagus, thymus, recurrent laryngeal nerve, strap muscles. * Pearson’s χ^2^ test or Fisher’s exact test, when appropriate. *p* < 0.05 was considered statistically significant.

**Table 6:** Pathological diagnosis changes after revision according to WHO 2022 criteria.

	Histological Diagnosis at the Time of Surgery	Histological Diagnosis According to WHO 2022 Criteria	*p* *
Atypical parathyroid tumour	135 (64.3)	140 (66.7)	0.251
Parathyroid carcinoma	75 (35.7)	70 (33.3)	

Data are given as absolute values with frequencies in brackets. * Wilcoxon signed—rank test. *p* < 0.05 was considered statistically significant.

**Table 7:** Post-operative complications.

	APT N = 226 (65)	PC N = 125 (35)	*p* *
Transient hypoparathyroidism	50 (22.3)	41 (33.3)	0.026
Permanent hypoparathyroidism	9 (4.1)	10 (8.3)	0.138
Transient unilateral recurrent laryngeal nerve palsy	8 (3.6)	7 (5.8)	0.409
Permanent unilateral recurrent laryngeal nerve palsy	3 (1.4)	10 (8.3)	0.002

Data are given as absolute values with frequencies (calculated on available data) in brackets. APT atypical parathyroid tumour; PC parathyroid carcinoma. * Pearson’s χ^2^ test or Fisher’s exact test, when appropriate. *p* < 0.05 was considered statistically significant.

## Data Availability

The datasets generated and analysed during the current study are available from the corresponding author upon reasonable request.
